# Biological Magnetic Resonance Data Bank

**DOI:** 10.1093/nar/gkac1050

**Published:** 2022-12-07

**Authors:** Jeffrey C Hoch, Kumaran Baskaran, Harrison Burr, John Chin, Hamid R Eghbalnia, Toshimichi Fujiwara, Michael R Gryk, Takeshi Iwata, Chojiro Kojima, Genji Kurisu, Dmitri Maziuk, Yohei Miyanoiri, Jonathan R Wedell, Colin Wilburn, Hongyang Yao, Masashi Yokochi

**Affiliations:** Department of Molecular Biology and Biophysics, UConn Health, Farmington, CT 06030-3305, USA; Department of Molecular Biology and Biophysics, UConn Health, Farmington, CT 06030-3305, USA; Department of Molecular Biology and Biophysics, UConn Health, Farmington, CT 06030-3305, USA; Department of Molecular Biology and Biophysics, UConn Health, Farmington, CT 06030-3305, USA; Department of Molecular Biology and Biophysics, UConn Health, Farmington, CT 06030-3305, USA; Protein Data Bank Japan, Institute for Protein Research, Osaka University, Suita, Osaka 565-0871. Japan; Department of Molecular Biology and Biophysics, UConn Health, Farmington, CT 06030-3305, USA; Protein Data Bank Japan, Institute for Protein Research, Osaka University, Suita, Osaka 565-0871. Japan; Protein Data Bank Japan, Institute for Protein Research, Osaka University, Suita, Osaka 565-0871. Japan; Graduate School of Engineering Science, Yokohama National University, Yokohama 240-8501, Japan; Protein Data Bank Japan, Institute for Protein Research, Osaka University, Suita, Osaka 565-0871. Japan; Department of Molecular Biology and Biophysics, UConn Health, Farmington, CT 06030-3305, USA; Protein Data Bank Japan, Institute for Protein Research, Osaka University, Suita, Osaka 565-0871. Japan; Department of Molecular Biology and Biophysics, UConn Health, Farmington, CT 06030-3305, USA; Department of Molecular Biology and Biophysics, UConn Health, Farmington, CT 06030-3305, USA; Department of Molecular Biology and Biophysics, UConn Health, Farmington, CT 06030-3305, USA; Protein Data Bank Japan, Institute for Protein Research, Osaka University, Suita, Osaka 565-0871. Japan

## Abstract

The Biological Magnetic Resonance Data Bank (BMRB, https://bmrb.io) is the international open data repository for biomolecular nuclear magnetic resonance (NMR) data. Comprised of both empirical and derived data, BMRB has applications in the study of biomacromolecular structure and dynamics, biomolecular interactions, drug discovery, intrinsically disordered proteins, natural products, biomarkers, and metabolomics. Advances including GHz-class NMR instruments, national and trans-national NMR cyberinfrastructure, hybrid structural biology methods and machine learning are driving increases in the amount, type, and applications of NMR data in the biosciences. BMRB is a Core Archive and member of the World-wide Protein Data Bank (wwPDB).

## INTRODUCTION

NMR is one of the most versatile analytic methods for analyzing matter, using atomic nuclei as embedded reporters. Applications of NMR to biomolecular science include structure, dynamics, and interactions of biomacromolecules in the solid and liquid solution state, the identity and composition of complex mixtures of small molecule metabolites, and the structure of natural products. BMRB is the open, international online repository for all types of biomolecular NMR data.

BMRB was founded by John Markley and Eldon Ulrich at the University of Wisconsin in 1988. Initial depositions were made by curators manually entering chemical shifts and assignments extracted from publications. With the development of the Adit-NMR deposition system, the primary means of accessions became depositor-driven. In 2006, BMRB joined the World-wide Protein Data Bank (wwPDB) (1) as a member and Core Archive. BMRB facilitates accession and validation of NMR-based biomacromolecular structures deposited to PDB via the OneDep system. BMRBj operating from Osaka University processes depositions in addition to serving as a mirror site. Another mirror site operates from CERM (https://bmrb.cerm.unifi.it/), at the University of Florence, IT. In 2020, BMRB moved to UConn Health in Farmington, CT.

BMRB depositions are linked to corresponding entries in PDB, the European Nucleotide Archive (2) (EMBL-EBI), the National Center for Biotechnology Information (3) (NCBI) and UniProt (4).

In addition to collaborations with wwPDB member organizations RCSB (5), PDBe (6), PDBj, (7) and EMDB (8), BMRB has close collaborations with the National Center for Biomolecular NMR Data Processing and Analysis (9) (NMRbox.org), the Center for High-Throughput Computing and the University of Wisconsin-Madison, the Collaborative Computing Project for NMR (10) (CCPN), Rutgers University, and the University of Frankfurt.

BMRBj (7), formerly known as PDBj-BMRB at Osaka, Japan, is a satellite BMRB repository that accepts NMR experimental data via three deposition servers, (i) OneDep depositions containing NMR experimental data from Asian countries and regions, (ii) BMRBdep deposition server at Osaka and (iii) SMSDep deposition server, which accepts NMR derived structures of biologically interesting small molecules that are ineligible for OneDep deposition due to the limit on molecular size. 10% of BMRB entries have been processed at Osaka site since 2002 (7). The BMRB and BMRBj sites share annotation tools and communicate closely on issues, assuring uniformity of annotation quality using either site.

## MATERIALS AND METHODS

### Accessions

Data housed at the BMRB are deposited, archived, and disseminated in the NMR-STAR file format (11). NMR-STAR is a variant of the STAR file format (12–14) introduced by Hall *et al.* in the early 1990s. STAR supports both tabular data as well as key-value pairs and is the format in use by the PDB. STAR also provides for optional grouping and stacking of similar data elements in what are referred to as save frames. NMR-STAR uses IUPAC (15) atom nomenclature. BMRB also supports the NMR Exchange Format (NEF) (16), which is also a variant of the STAR format, and conforms to definitions from the Collaborative Computing Project for NMR (CCPN) data model (10).

The BMRB maintains the NMR-STAR data dictionary, the details of which can be found on the website (https://bmrb.io/dictionary/). The current dictionary version (3.2) consists of more than 6500 defined data tags organized across >100 save frame categories corresponding to eight super-groups. These data definitions cover the various types of data archived at the BMRB: chemical shifts, relaxation, and hydrogen exchange rates (including nuclear Overhauser effects, NOE’s, and scalar or dipolar coupling constants). They also cover the various types of metadata supporting the depositions: molecular assembly definitions, sample conditions and experiment setup, author information, along with cross-references to other databases of interest. The NMR-STAR dictionary is also used by external software tools which read and/or write NMR-STAR data.

The hierarchical nature of the STAR file format makes it suitable for representation as XML or JSON. A generic XML schema definition has been proposed for STAR files in general (17); the BMRB also disseminates entries in other formats conforming to a schema specific for the current version of the NMR-STAR data dictionary (18).

Whereas the content of the master BMRB archive and BMRBj are identical, BMRBj has explored alternative approaches to enhance reusability and interoperability of NMR experimental data using open standard and community-driven technologies. One example is to provide NMR experimental data in web standard formats, XML and RDF (https://www.w3.org/TR/rdf11-concepts/). This extended archive is now also available from the main BMRB web site (18). Based on the extended archive, BMRBj launched a portal site that can search BMRB and related databases simultaneously and display NMR experimental data as rich graphical content (19). BMRBj is leading the effort to remediate legacy depositions of NMR restraints in PDB, converting software-specific formats to the community-based NEF format.

### Deposition systems

**Figure 1.**
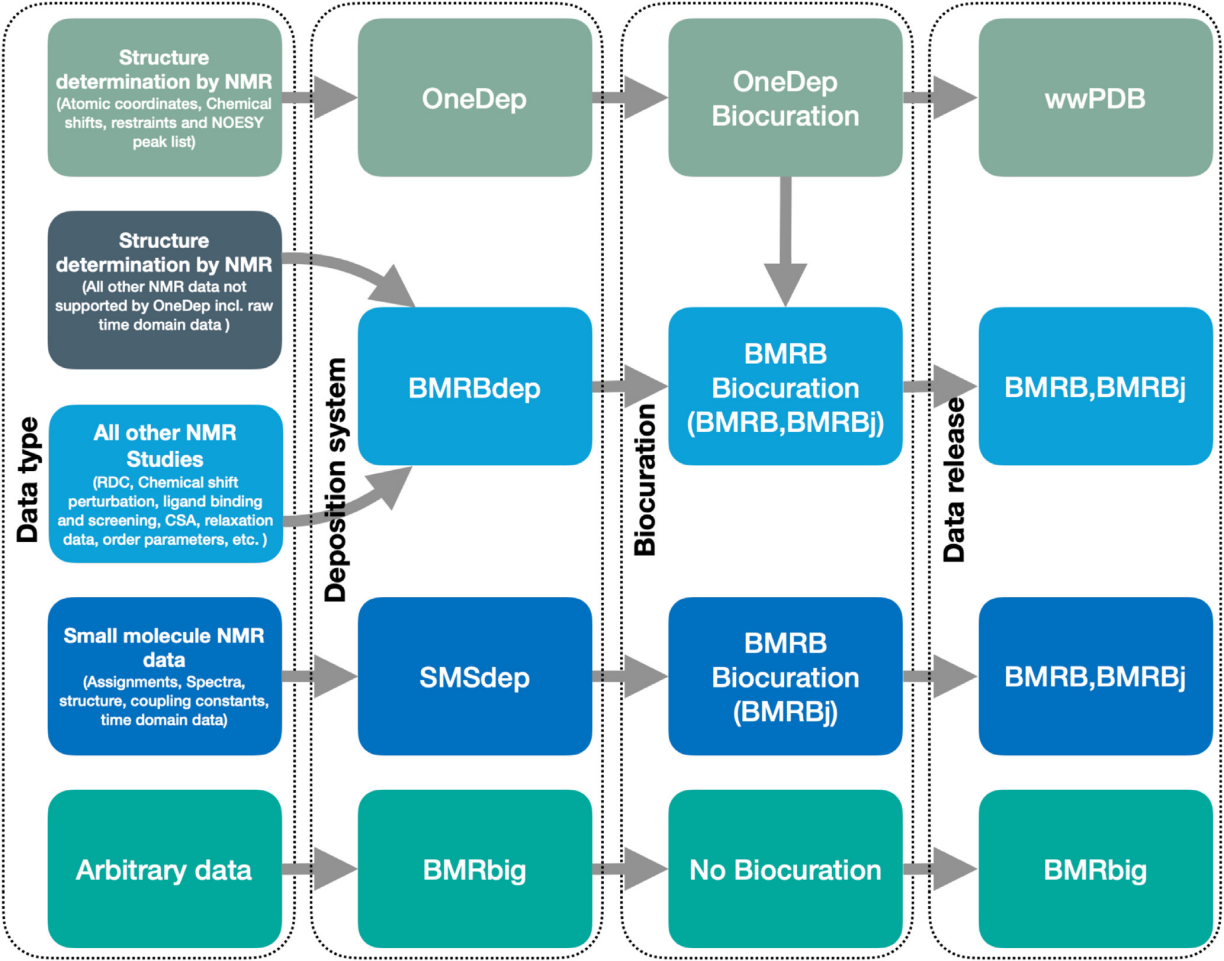
BMRB supports deposition through OneDep, BMRBdep, SMSdep and BMRBbig.

BMRB currently operates four distinct deposition systems, each with a specific role (Figure 1). OneDep (20) is the system jointly managed with the wwPDB for accession of NMR data in support of a biomacromolecular structure, typically proteins or nucleic acids. It accepts atomic coordinates, chemical shifts, geometrical constraints, and nuclear Overhauser effect (NOESY) peak lists. BMRBdep (21) is the deposition system for NMR data not associated with a PDB structure deposition. It accepts data for systems that do not meet the size threshold for PDB, or include data that is not supported by OneDep, for example raw time-domain NMR data. SMSDep is the deposition system for biomolecule structures that do not fit the OneDep criteria. BMRB also operates a generic, uncurated deposition system that accepts arbitrary data, BMRbig (bmrbig.org).

### BMRBdep

BMRBdep is the internally developed and managed system for capturing NMR data that is not associated with a PDB structure deposition. BMRBdep is a SPA (single page application) developed using the modern Angular web application framework. It supports *de novo* depositions, using an existing deposition as a starting point, or bootstrapping a deposition via the upload of an NMR-STAR file. In addition, it supports ‘off-line’ deposition work - allowing deposition form to be filled even if internet access is temporarily unavailable, and saving the changes when connectivity is restored. Another notable feature is the ability to ‘clone’ a deposition at any point during the deposition process, which is helpful when depositing multiple datasets that share some metadata.

BMRBdep was designed to allow for future enhancements such as the ability for a depositor to access a ‘profile’ page of all their depositions, as well as the ability to grant access to other co-depositors or grant limited read-only access to journal reviewers prior to release. After deposition, data from BMRBdep is entered into the common BMRB annotation process which is also used for OneDep and SMSDep depositions.

### OneDep

The chemical shifts, restraints, and peak lists from structure determination studies are currently deposited through the OneDep (https://deposit.wwpdb.org) system. OneDep is a unified system for deposition, biocuration, and validation of experimentally determined structures of biological macromolecules to the PDB archive. It maintained as a global collaboration by the wwPDB partners (RCSB-PDB, PDBe, PDBj, BMRB and EMDB). BMRB developed the biocuration and validation protocol for the NMR data for the OneDep system.

The OneDep system currently accepts all the NMR experimental data either in NMR-STAR v3.2 or in NMR Exchange Format (NEF) v1.2. The legacy method of uploading chemical shifts in NMR-STAR format, with restraints and peak lists in any software-specific format will be phased out, tentatively by the end of 2023. The OneDep system shares the NMR data after initial bio curation and validation with BMRB for further annotation. BMRB adds additional metadata including author and experimental details, sample conditions, etc. to the deposition and prepares a stand-alone NMR-STAR file for the BMRB archive. The wwPDB releases most of the metadata along with the structure coordinate data in CIF (22) format and experimental data with minimal metadata in NMR-STAR format. The data will be released simultaneously by both the PDB and BMRB archives subsequent to a release request from the depositor.

WwPDB has a data deposition policy that specifies certain minimal sizes for biopolymers (https://www.wwpdb.org/documentation/policy). Crystal structures of peptides with fewer than 24 residues within any polymer chain that do not meet the criteria wwPDB criteria should be deposited at the Cambridge Crystallographic Data Centre (23) (CCDC, http://www.ccdc.cam.ac.uk/products/csd/deposit/). NMR structures of such molecules should be submitted to BMRB through SMSDep (http://smsdep.protein.osaka-u.ac.jp/bmrb-adit/).

### SMSDep

SMSDep was developed by BMRB for biomolecular structures that cannot be deposited via OneDep, in particular: peptides with fewer than 24 residues in any polymer chain. SMSDep system is operated by BMRB-Japan (BMRBj), with structure validation carried out by PDBj and NMR data curation by BMRBj. SMSDep is the only option available for depositing experimentally determined structures of small peptides from NMR studies.

### Content

The BMRB archive is dominated by assigned chemical shifts (frequencies of nuclear resonances) for ^1^H, ^13^C and ^15^N nuclei in proteins and nucleic acids. There are currently >10 868 000 assigned chemical shifts corresponding to 15451 studies in the archive. Additional data includes nuclear magnetic relaxation rates, hydrogen exchange rates, and scalar internuclear coupling constants. There are experimental time-domain data sets and experimental reference spectra for more than 1200 common small-molecule metabolites. The BMRB archive includes a richly annotated collection of small molecule entries that are uniquely identified by ALATIS-derived atom nomenclature IDs. Additionally, more than 650 standard small-molecule spectra are complemented by GISSMO-derived spin system matrices, thus enabling 1D NMR spectral simulation at arbitrary spectrometer field strengths.

### Shift statistics

**Figure 2.**
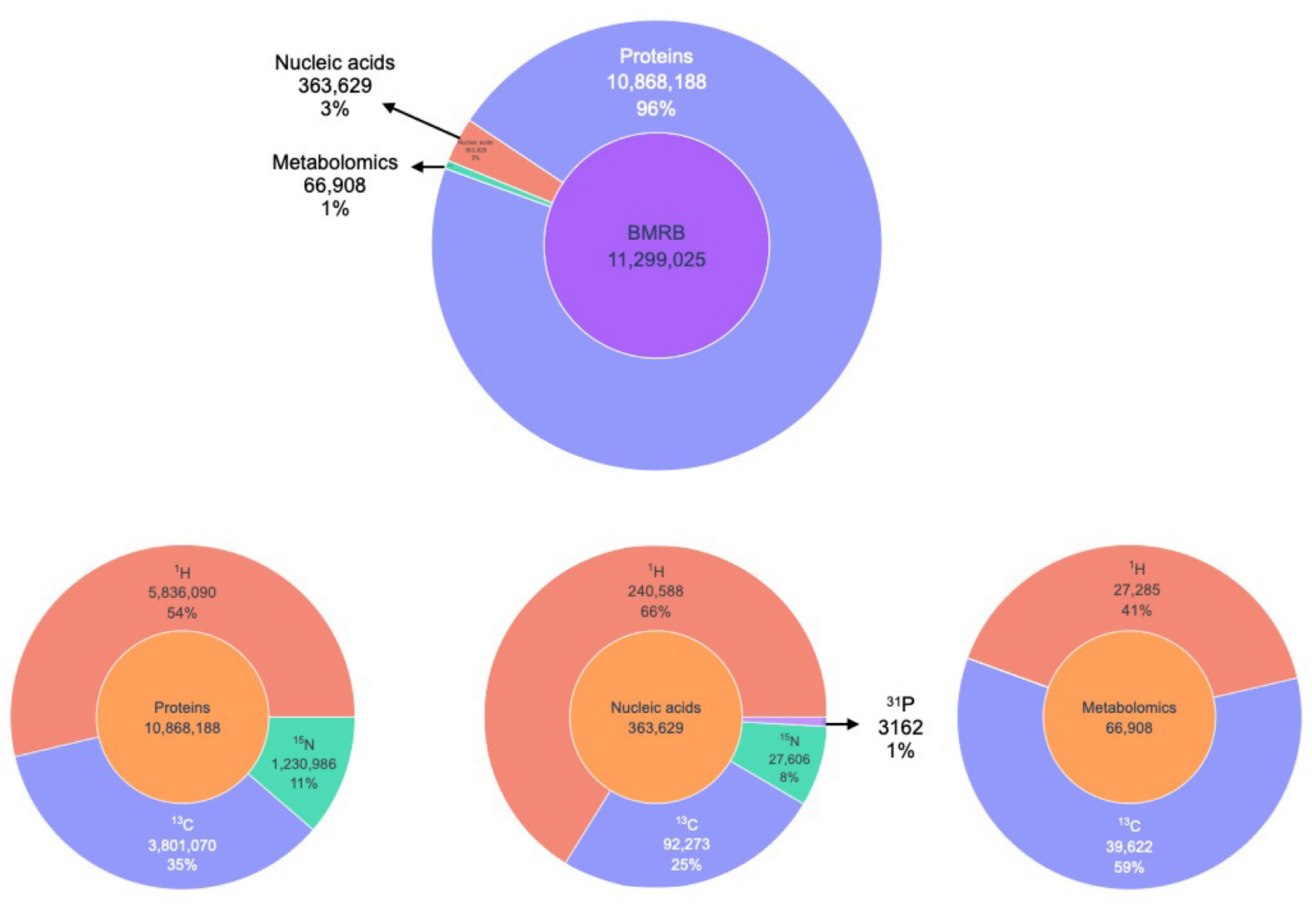
Chemical shift statistics for BMRB as of 14 September 2022. The chemical shifts for nuclei other than ^1^H, ^13^C, ^15^N and ^31^P are included in the totals, but not shown in the chart because of their small contribution.

BMRB hosts more than 11 million experimentally measured chemical shifts from biologically important molecules including proteins, nucleic acids and metabolites. Figure 2 depicts the chemical shifts content of BMRB by molecule and atom type. Up-to-date and detailed statistics on content of BMRB in tablular form is available from the ‘Query grid’ link on the search page at https://bmrb.io/search. Other pre-computed query data is also available.

### Services

In addition to data accessions via the OneDep (for biomacromolecular structures determined by NMR), BMRBdep (for biomolecule NMR data not associated with a macromolecular structure), and SMSDep (for small molecules) services, BMRB provides a web-based service for querying the BMRB archive, application programming interfaces (APIs) for querying the archive and manipulating NMR data in NMR-STAR format, and endpoints for batch downloads of the archive. Snapshots of the archive are maintained on the NMRbox (nmrbox.org) platform. BMRB also provides a CS-Rosetta service for determining protein structure from NMR data via a dedicated high-performance computing cluster. The BMRB web site also provides rich statistics on the archive content, documentation of NMR data standards, and reference and educational resources related to biomolecular NMR. The BMRbig (bmrbig.org) platform provides a persistent archive for arbitrary data on biomolecules, and serves as a transitional repository enroute to full BMRB deposition. BMRB also hosts the ALATIS server (https://alatis.bmrb.io) for generating unique INCHI IDs. BMRB provides software resources through the GitHub (https://github.com/bmrb-io) repository for developers. The source code for the NMR-STAR parser (PyNMRSTAR : https://pypi.org/project/pynmrstar/) and data visualization tools in Python(PyBMRB: https://pypi.org/project/pybmrb/)and R(RBMRB https://CRAN.R-project.org/package=RBMRB) are available through BMRB GitHub.

### Curated data types

The BMRB archive accepts arbitrary data types, however most major types of biomolecular NMR data are validated as part of the curation process. The most important are assigned chemical shifts, i.e. the nuclear resonance frequencies of specific atoms in the biomolecule. Other types include distance restraints between hydrogen atoms, derived from nuclear Overhauser effects (NOE); nuclear relaxation rates including spin-lattice (R_1_), spin-spin (R_2_) and heteronuclear NOE; and unassigned chemical shifts (as peak tables in NMR-STAR or text format).

### Accessing content

BMRB services are primarily accessed through the BMRB web site (https://bmrb.io—see next section) but BMRB data is made available in a variety of ways to facilitate community access. These support both interactive use as well as programmatic access. For programmatic access, BMRB provides data via an API, an rsync endpoint, a Globus endpoint, and via ReBoxitory. The BMRB API supports programmatic queries against the BMRB database. Querying for entries based on tag values (searches) is supported, as is looking up corresponding entries from related databases (PDB entries, UniProt entries), searching through the chemical shifts, searching for structures based on a set of chemical shifts, and many other queries. (https://github.com/bmrb-io/BMRB-API)

Additional bulk access to data (both NMR-STAR data, as well as primary data such as time domain data) is supported via rsync and Globus endpoints. Both rsync and Globus are software tools that enable a user to select one or more folders of data to synchronize between a local computer and the BMRB archive. rsync requires a manual step to update the local copy of data, whereas Globus can keep data synchronized continuously. rsync is a standard Unix tool and is included by default on nearly all Linux and Mac operating systems (though also available on Windows). Globus requires downloading the Globus utilities and creating an account before accessing BMRB data, but it is commonly used in academic settings for accessing and transferring data, and as such many researchers already have the Globus software installed and configured. Within the Globus software, searching for ‘BMRB’ will return the available BMRB data sets within the Globus network. More details on using rsync or Globus to access BMRB data are available on the BMRB web site (https://bmrb.io/data_library/rsync.shtml, https://bmrb.io/data_library/globus.shtml)

Another means of data access is via ReBoxitory - a ‘data lake’ provided automatically to all users of the NMRbox Platform as a Service (PaaS). ReBoxitory provides versioned copies of multiple public domain databases (BMRB, PDB, AlphaFold, etc.) via the file system within NMRbox. NMRbox users can simply browse to */reboxitory/$year/$month/$database* to access the data. For BMRB, the data is available in the ‘BMRB’ folder. $year and $month are variables which should be substituted with a 4-digit year and 2-digit month to access a given snapshot of the data. Available data includes the entire macromolecule and small molecule archives in NMR-STAR format, any corresponding raw data, as well as dumps of the relational database tables in use by BMRB for these databases.

### Web site

The BMRB web site underwent a major modernization project in 2020, the results of which are now public. The redesigned site includes a significantly improved navigation panel which takes up less space on the screen and also reorganizes content for easier navigation according to intuitive categories, has a mobile version of the navigation that automatically displays on mobile devices, loads faster, and has a new home page designed to help users discover available BMRB services. As part of this modernization, the BMRB has also moved from its traditional bmrb.wisc.edu domain to the new bmrb.io domain and has rebranded with a much more modern and heuristic logo that matches the redesign of the BMRB web site.

Primary changes that users will notice are the new design, the new home page which highlights primary BMRB resources in a tiled grid, and the improved navigation menu. The primary sections of the new BMRB menu help different types of users (from students to established domain experts) to quickly locate the resources they are looking for. The new primary menu categories are About, Deposit, Search, Visualize, Analyze, Data and Learn. When these menu categories are highlighted (or selected, in the case of a mobile device) they expand to show the various resources available within that category. Furthermore, resources can be categorized under additional headers within these categories, such as the various links in the ‘Data preparation’ section of the Deposit category.

Despite the major improvements to the navigation and appearance of the site, no legacy URLs were broken in the process of upgrading the site. This means that bookmarks and published links to BMRB were operative during the update process.

In addition to the site redesign, BMRB is continually working on new features, such as an improved deposition statistics page (https://bmrb.io/bmrb/deposition_stats.shtml) and an interactive map showing the geographic distribution of BMRB entries (https://bmrb.io/bmrb/deposition-map.shtml). User suggestions are welcomed at any time, and can be made by clicking the ‘Support’ button on the lower right corner of the page and submitting a request to BMRB staff.

**Figure 3.**
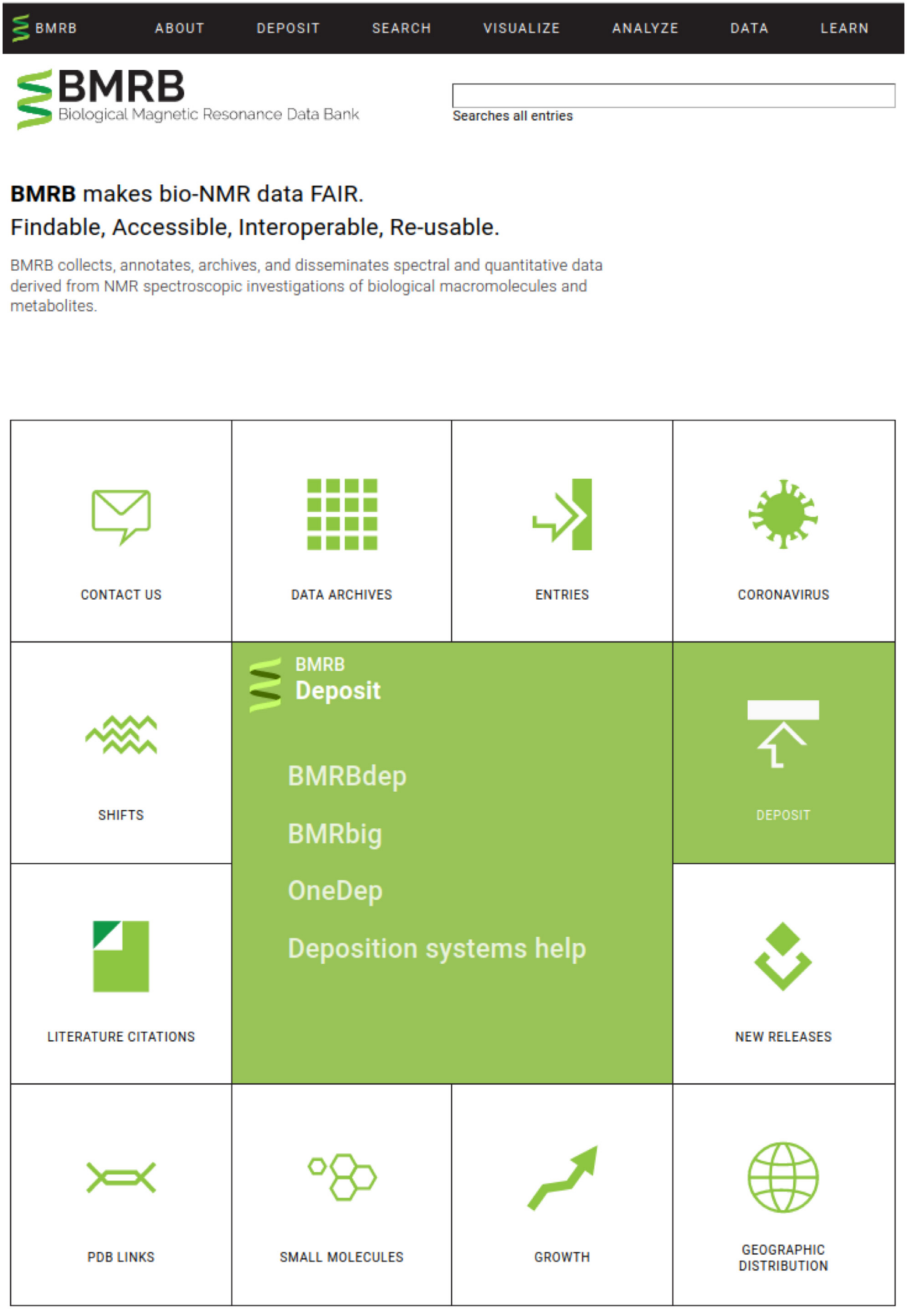
Landing/home page for BMRB.

Figure 3 depicts the new home page of the BMRB, showing the revised navigation menu as well as the new home page grid. Here the ‘Deposit’ section of the grid has been clicked, which has updated the center tile to show information on available BMRB deposition options.

### Small molecules

BMRB has been the repository of record for small molecule structure data that do not satisfy the PDB structure deposition criteria - for example, oligopeptides with less than 24 residues. In recent years, BMRB has become a pioneering repository for spectra of small molecule by incorporating several innovations. The current archive of standards contains 1343 small molecules comprising of 656 metabolites and 665 fragments from the Maybridge fragment library. More than 1200 compounds, includes many key mammalian metabolites and drug-like molecular fragments used in ligand screening are represented. Small molecule data includes theoretically derived chemical shifts for 219 compounds. Additionally, BMRB contains between one to three sets of chemical shifts, corresponding to different conditions, for 487 lignins. BMRB is undertaking additional innovations to improve the quality and interoperability of open data related to small molecules, such as metabolites, drugs, natural products, food additives, and environmental contaminants. Small molecules are provided a unique identifier based on the implementation of an extended version of the IUPAC International Chemical Identifier (InChI) system called ALATIS (24,25) (https://alatis.bmrb.io/). The implementation utilizes the three-dimensional structure of a compound to generate reproducible compound identifiers (standard InChI strings) and universally reproducible designators for all constituent atoms of each compound. Another factor that has contributed to improved quality is the GISSMO (26,27) mechanism for the specification and exchange of small molecule NMR data in a robust way by parameterizing the description of the experimental data.

ALATIS addresses the critical problem of deriving a complete, consistent, and unique representation for describing a compound based on its 3D structure in a format that can be sorted and compared. During this process the compound itself and all its constituent atoms will receive unique and reproducible names – including the protons. ALATIS uses an automated algorithm for the bijective mapping between the descriptor and 3D structure to avoid human error, and it also maintain a low time complexity order in order to mitigate very long computation times for molecules with a large number of atoms. The algorithm generates a standard InChI string as the unique identifier and assigns unique atom identifiers (numbers) to each atom in the molecule. Because of this unique mapping, the software can also take this enhanced InChI string and generate a 3D model for the compound with all atoms reproducibly identified.

GISSMO (https://gissmo.bmrb.io) provides a mechanism for the specification and exchange of NMR data in a robust way by parameterizing the description of the experimental data. Parameterization of experimental data is specialized work that entails expert modelling and detailed specification, but it is another key necessary step for advancing data quality and usability. Associated NMR-STAR meta-data for each entry serves to define the calculated parameters and associates them with computer-readable information describing data collection, such as sample properties (concentration, pH, temperature, buffer), NMR instrument manufacturer, model, field-strength, type of probe, etc. The quality of the derived spin system model is evaluated by calculating the RMSD between the experimental spectrum and the spectrum calculated from the spin system at the corresponding magnetic field.

The website for GISSMO maintains tools for simulating 1H NMR spectra of mixtures of small molecules and for automated peak analysis of 1H NMR spectra of biological fluids or cell extracts to identify compounds present. Once the spin system matrix has been accurately modelled to represent an experimental NMR spectrum collected at a given magnet field strength, the matrix can be used to model spectra at any desired magnetic field strength. The ^1^H NMR spectrum of each compound is simulated at a variety of magnetic fields (40, 60, 80, 90, 100, 200, 300, 400, 500, 600, 700, 750, 800, 900, 950, 1000, 1100 and 1300 MHz). Every entry modelled by the GISSMO modelling process can be downloaded in NMR-STAR and NMReDATA data format.

The search functions of BMRB, available under the ‘Search’ menu item in the top banner of the website, includes a peak query function under the heading ‘chemical shift search’. Peak query is a recent service provided by the BMRB that has garnered significant usage by the community. The web form allows the user to enter a list of ^13^C, ^15^N or ^1^H chemical shift values. The values are used to search entries, with the highest priority given to the match with the largest number of peaks, and the second highest priority to the closeness of the matches in ppm. The service is available for both metabolomics and macromolecular entries.

### Educational/reference

The BMRB web site hosts an extensive collection of documents defining standards for nomenclature, chemical shift referencing, and data formats, as well as background tutorials on biomolecular NMR. It also provides many links to external web services pertinent to biomolecular NMR.

### CS-Rosetta server

BMRB developed and released a CS-Rosetta (28) service in 2010 and has continued to maintain and improve it. Until 2022, the service used the computational resources of the CHTC (Center for High Throughput Computing) and OSG (Open Science Grid), when it was migrated to a dedicated cluster at UConn. The service accepts chemical shifts in either NMR-STAR or TALOS format and permits selection of the number of structures to generate. The server then uses the HTCondor (https://htcondor.org) high throughput computing software to distribute the workload across a large number of machines and computing cores. This massive parallelism has allowed BMRB to provide results in <24 h, even for large numbers of structures. With recent improvements and a dedicated computational cluster, results are now provided in as little as 4 hours.

While the standard submission form supports options such as whether or not to remove flexible tails, whether any disulfide bonds are present, and whether or not to exclude homologous structures, the BMRB CS-Rosetta server also provides an advanced submission mode that allows submission of constraints (NOE or otherwise derived) in the Rosetta constraints format, and residual dipolar couplings (RDCs).

### BMRbig

BMRbig is a BMRB project designed to accommodate the acquisition of diverse data (not just NMR data) beyond the types currently curated and annotated by BMRB. Unlike BMRBdep, it allows for the deposition of arbitrary data and collects the appropriate metadata provided by the depositor. To facilitate timely release of data into the public domain, BMRbig provides the ability for depositors to release data nearly immediately without undergoing a lengthy annotation process. It also supports updating deposited data, and appending additional data to existing depositions. A versioned history is maintained of all changes, and future improvements will allow reviewing any particular release of a BMRbig entry.

## RESULTS

BMRB has enabled the development of dozens of software packages that are used by thousands of investigators every day. These include TALOS and PECAN, for determining secondary structure of proteins; Sparta (29), ShiftX2 (30), CAMSHIFT, UCBShift (29) and 4DSPOT (30) for prediction of chemical shifts from protein structure; Chemical Shift Index, for identifying disordered regions of proteins; CS-Rosetta (28,30) and CHESHIRE (31) for determining protein structures from chemical shifts; CCPN, for the analysis of protein NMR spectra; FLYA (32) and PINE (33), for assignment of protein NMR spectra; CYANA (34) and XPLOR-NIH (35). for computing macromolecular structures from NMR data; LACS and PANAV for referencing protein chemical shifts against standards. Collectively, the publications describing these software packages have been cited >16 000 times (Google Scholar).

As BMRB grows, the utility of the archive extends beyond discerning general trends to identifying unusual structural or dynamical features of biomacromolecules. BMRB has crossed the threshold where extreme values no longer are merely statistical outliers, but now are abundant and capable of revealing hidden properties. A recent example (36) is an investigation of shift outliers as sentinels of hydrogen bonds between amide N–H groups (H-donor) and aromatic rings (H-acceptor). First postulated in proteins by Perutz and Levitt (37), federation of BMRB chemical shift data with PDB structures revealed evidence of hundreds of amide-aromatic H-bonds.

Although BMRB content is dominated by protein data, applications of NMR to RNA are increasingly important. Examples include applications to hybrid/integrative structural biology (38) and investigating RNA as potential drug targets (39).

## DISCUSSION

Emerging and future applications of BMRB will enable deeper insights into biomacromolecular dynamics and disorder, including LLPS (liquid-liquid phase separation) propensity, dynamics from relaxation rates and chemical shifts, conformational propensities of disordered proteins and nucleic acids, the detection and characterization of binding interactions (37,39,40), personalized medicine (41) (drug metabolism and pharmacokinetics, diagnostics), and microbial metabolomics (42–45). As a rich source of curated data, BMRB will foster the application of advances in machine learning (ML) to these challenge areas. BMRB was an early driver of applications of neural networks to biomolecular NMR (46–48). Curated biomolecular NMR data is essential for realizing the potential of deep learning for unlocking latent knowledge present in biomolecular NMR data.

## DATA AVAILABILITY

The URL for BMRB is https://bmrb.io. BMRB maintains an open-source GitHub repository (https://github.com/bmrb-io).
